# Early correction of base deficit decreases late mortality in polytrauma

**DOI:** 10.1007/s00068-022-02174-9

**Published:** 2022-11-22

**Authors:** Karlijn J. P. van Wessem, Falco Hietbrink, Luke P. H. Leenen

**Affiliations:** Department of Trauma Surgery, University Medical Center Utrecht, Heidelberglaan 100, 3584 CX Utrecht, The Netherlands

**Keywords:** Polytrauma, Physiological derangement, Mortality

## Abstract

**Introduction:**

Physiology-driven resuscitation has become the standard of care in severely injured patients. This has resulted in a decrease in acute deaths by hemorrhagic shock. With increased survival from hemorrhage, focus shifts towards death later during hospital stay. This population based cohort study investigated the association of initial physiology derangement correction and (late) mortality.

**Methods:**

Consecutive polytrauma patients aged > 15 years with deranged physiology who were admitted to a level-1 trauma center intensive care unit (ICU) from 2015 to 2021, and requiring surgical intervention < 24 h were included. Patients who acutely (< 48 h) died were excluded. Demographics, treatment, and outcome parameters were analyzed. Physiology was monitored by serial base deficits (BD) during the first 48 h. Correction of physiology was defined as BD return to normal values. Area under the curve (AUC) of BD in time was used as measurement for the correction of physiological derangement and related to mortality 3–6 days (early), and > 7 days (late).

**Results:**

Two hundred thirty-five patients were included with a median age of 44 years (70% male), and Injury Severity Score (ISS) of 33. Mortality rate was 16% (71% due to traumatic brain injury (TBI)). Median time to death was 11 (6–17) days; 71% died > 7 days after injury. There was no difference between the single base deficit measurements in the emergency department(ED), operating room (OR), nor ICU between patients who died and those who did not. However, patients who later died were more acidotic at 24 and 48 h after arrival, and had a higher AUC of BD in time. This was independent of time and cause of death.

**Conclusion:**

Early physiological restoration based on serial BD measurements in the first 48 h after injury decreases late mortality.

**Supplementary Information:**

The online version contains supplementary material available at 10.1007/s00068-022-02174-9.

## Introduction

Physiology-driven resuscitation has become standard of care in severely injured patients. This has caused a decrease in acute deaths by hemorrhagic shock resulting in both a delayed time to death, and an increased proportion of death by other causes than exsanguination [1]. Recent studies have demonstrated that traumatic brain injury (TBI) has become the most common cause of death in blunt traumatic injury in the western world with up to 77% of death caused by TBI [1–6]. Besides prevention of brain injury, treatment should focus on preventing secondary damage of the brain once the injury has occurred. One of these strategies is prompt and adequate restoration of physiological derangement. Measurements such as heart rate, blood pressure, and hemoglobin have shown to be inaccurate for identifying severe hemorrhage and predicting outcome [7–10]. However, indicators of anaerobic metabolism and acidosis, reported as being strongly associated with the degree of blood loss and outcome, have been suggested as end points of resuscitation [7, 11, 12].

One of the parameters that represent acidosis is base deficit (BD) which has been regarded as a reliable parameter of physiological derangement, and a deeper arterial BD has been consistently associated with increased mortality [11–19]. However, in most reports no distinction was made between acute (< 48 h), early (3–6 days) and late (> 7 days) deaths. Additionally, only few studies have reported serial base deficit measurements during hospital stay [12, 19].

We hypothesized that early and adequate restoration of physiological derangement could beneficially influence both early and late mortality in a polytrauma population in which TBI has become the main cause of death. Therefore, we conducted a retrospective cohort study with prospectively collected data to investigate whether early correction (within 48 h) of physiological derangement measured by serial base deficits up to 48 h after injury would be related to death later than 48 h in severely injured patients.

## Materials and methods

A prospective population-based cohort study was undertaken to investigate outcomes in severely injured patients admitted to the intensive care unit (ICU) of a major (Level-1) trauma center (University Medical Center Utrecht, the Netherlands). Details of the hospital and catchment area were previously described [20]. From January 2015 until December 2021 all consecutive polytrauma (Injury Severity Score (ISS) > 15) patients > 15 years of age who had a deranged physiology (defined as base deficit < − 2.0 mEq/L), underwent urgent surgery (< 24 h after admission) and were admitted to the adult ICU were included. Patients who died within 48 h after injury were excluded. Additionally, patients who were referred from other institutes later than 24 h after injury were excluded as well. Patients with isolated TBI, asphyxiation, drowning and burns were also excluded, because of potential different physiologic response to severe trauma and a significantly different mortality and morbidity profile [21, 22]. Isolated injury to the brain was defined as Abbreviated Injury Scale (AIS) head > 3 and AIS < 2 in other regions.

All data were prospectively collected by authors KW and LL and included demographics, shock and resuscitation parameters. Administration of both crystalloid and blood products was documented in the first 24 h after admission. Further, detailed data of all surgical interventions per patient within the first 48 h after admission were documented and contained type, timing and duration of surgery (total time in Operation Room (OR)), per-operative physiology (base deficit, hemoglobin, temperature) and resuscitation parameters (crystalloids, packed red blood cells (PRBC), fresh frozen plasma (FFP), platelets (PLT), tranexamic acid (TXA)). Additionally, Denver Multiple Organ Failure (MOF) scores [19], and Adult Respiratory Distress Syndrome (ARDS) Berlin criteria [23] were registered daily up until 28 days or discharge from ICU.

Correction of physiological derangement was measured by arterial base deficit which was repeatedly measured until normalization (BD > − 2.0 mEq/L) or up until 48 h after arrival. Arterial base deficit was directly calculated from the blood gas analyzer from the partial pressure of carbon dioxide in arterial blood (PaCO_2_), pH, and serum bicarbonate (HCO_3_) as applied to a standard nomogram and represents the number of milli-equivalents of additional base that must be added to a liter of blood to normalize pH. Base deficit was chosen to measure physiological derangement since it has proven to be a consistent predictor of the need for hemostatic resuscitation and mortality in severely injured patients [11–18]. In all patients BD (worst value in case of several measurements) was measured in the Emergency Department (ED, *t *= 0), OR, and on arrival in ICU. Additional BD after 24 h and 48 h after arrival was measured in case of ongoing physiological derangement.

Area under the curve (AUC) was used as measurement for the correction of physiological derangement during the first 48 h. AUC defined as the whole area under the curve was an integrated measurement of changes of base deficit in time. Any peak whose height was less than 5% of the distance from minimum to maximum Y value was ignored.

Primary outcome was the evaluation of the relation between the timing of physiological derangement correction in the first 48 h and both early (3–6 days) and late (> 7 days) mortality.

### Ethical approval

The local ethics committee approved this prospective observational study and waived consent (reference number WAG/mb/16/026664).

### Statistical analysis

Data were analyzed using IBM SPSS Statistics, version 26.0 (Armonk, NY, USA). GraphPad Prism version 9.3.0 (San Diego, CA, USA) was used to calculate AUC and to prepare the graphs. Results are presented as medians with interquartile range (IQR). Comparison of continuous variables was done using Kruskal–Wallis. Significant differences for categorical variables were calculated through Chi-square test or Fisher’s exact test depending on the size of the groups. Receiver-operator characteristic (ROC) curve was used to evaluate the performance of AUC in prediction of mortality. Variables with univariate statistical significance of less than 0.10 were included in a multivariate logistic regression analysis. These variables were analyzed with forward stepwise selection to identify independent risk factors for mortality and presented as odds ratios and 95% confidence intervals. Statistical significance was defined as *P* < 0.05.

## Results

**Fig. 1:**
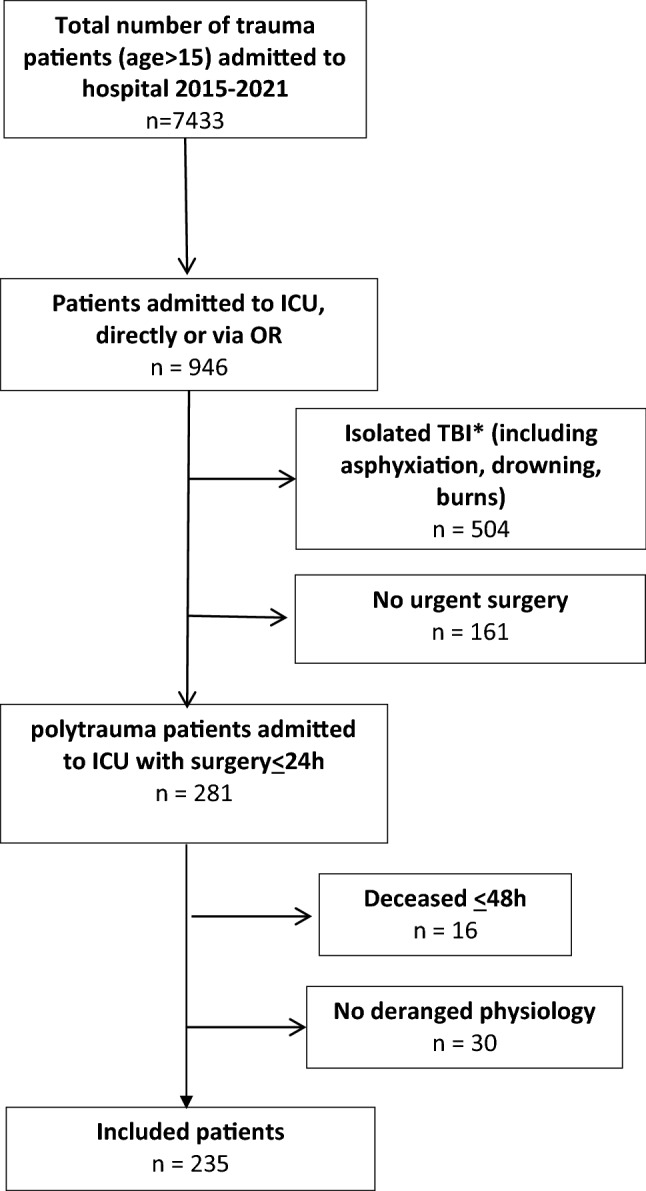
Flowchart of included patients. *Isolated traumatic brain injury (TBI) was defined as Abbreviated Injury Score (AIS) head > 3 and AIS < 2 or less in other regions

Two hundred thirty-five severely injured patients (70% male) with a median age of 44 (28–58) years with deranged physiology who underwent urgent surgery (< 24 h) and were admitted to ICU were included. A flowchart of included patients is shown in Fig. 1. Ninety-one percent of injuries (*n* = 213) were caused by a blunt mechanism and median Injury Severity Score (ISS) was 33 (24–38) with most severe injuries located in the brain (AIS head 3 (0–4)) and chest (AIS chest 3 (2–4)). Physiology, resuscitation, and outcome data are presented in Table 1.

**Table 1:** Demographics of polytrauma population who survived and died

	Total population (*n* = 235)	Survival (*n* = 197)	Mortality (*n* = 38)	*P* value
Age (years)	44 (28–58)	39 (26–56)	68 (40–73)	< 0.001*
Male gender	164 (70%)	135 (69%)	29 (76%)	0.34
Blunt MOI	213 (91%)	176 (89%)	37 (97%)	0.22
Prehospital intubation	91 (39%)	72 (37%)	19 (50%)	0.14
ISS	33 (24–38)	30 (24–38)	38 (27–43)	0.02*
AIS head	3 (0–4)	3 (0–4)	4 (2–5)	< 0.001*
AIS face	0 (0–1)	0 (0–1)	0 (0–2)	0.33
AIS chest	3 (2–4)	3 (2–4)	3 (2–4)	0.005*
AIS abdomen	2 (0–4)	2 (0–3)	2 (0–4)	0.09
AIS pelvis/extremities	2 (1–3)	2 (1–3)	2 (2–3)	0.28
AIS external	0 (0–1)	0 (0–1)	0 (0–1)	0.94
SBP_ED (mmHg)	114 (90–134)	114 (90–133)	116 (76–144)	0.97
SBP < 90 mmHg_ED	68 (29%)	53 (27%)	15 (40%)	0.12
Hb_ED (mmol/L)	7.8 (7.0–8.9)	7.8 (7.0–8.9)	7.8 (6.5–8.4)	0.37
pH_ED	7.30 (7.23–7.36)	7.30 (7.24–7.36)	7.26 (7.17–7.35)	0.047*
PaC02_ED (mmHg)	45 (40–52)	44 (39–52)	48 (42–55)	0.04*
BD _ED (mEq/L)	− 4.0 (− 7.0–2.0)	− 4.0 (− 7.0–2.0)	− 6.0 (− 9.0–2.3)	0.08
PT_ED (sec)	14.2 (13.0–16.4)	14.2 (12.9–16.1)	14.8 (13.5–18.5)	0.03*
Temperature_ED (^o^C)	35.5 (34.5–36.4)	35.5 (34.6–36.4)	34.9 (33.9–35.6)	0.09
Resuscitation parameters				
Crystalloids < 24 h (L)	8.7 (6.7–11.4)	8.3 (6.7–10.9)	11.3 (8.0–13.3)	0.003*
PRBC < 24 h (U)	4 (1–8)	4 (1–8)	4 (1–12)	0.42
PRBC > 10 units < 24 h	46 (20)	36 (18)	10 (26)	0.27
FFP < 24 h (U)	4 (0–9)	4 (0–9)	6 (0–14)	0.18
PLT < 24 h (U)^a^	0 (0–2)	0 (0–1)	1 (0–3)	0.29
TXA	191 (81%)	162 (82%)	29 (76%)	0.39
Outcome parameters				
Ventilator days	6 (3–11)	6 (2–11)	9 (5–15)	0.002*
Ventilator free days	14 (7–21)	16 (10–22)	0 (0–1)	< 0.001*
ICU LOS (days)	7 (3–14)	7 (3–14)	10 (5–15)	0.08
H-LOS (days)	23 (14–34)	25 (17–35)	11 (6–17)	< 0.001*
MODS	42 (18%)	26 (13%)	16 (42%)	< 0.001*
ARDS	6 (3%)	5 (3%)	1 (3%)	1.0
Infectious complications	114 (49%)	98 (50%)	16 (42%)	0.39

Data are expressed in median (IQR) or absolute numbers (%)

*MOI* mechanism of injury, *ISS* injury severity score, *AIS* abbreviated injury scale, *ED* emergency department, *SBP* systolic blood pressure, *Hb* hemoglobin, *PaC02* partial pressure of carbon dioxide in arterial blood, *BD* base deficit, *PT* prothrombin time, *PRBC* packed red blood cells, *FFP* fresh frozen plasma, *PLT* platelets, *TXA* tranexamic acid, *ICU* intensive care unit, *LOS* length of stay, *H-LOS* hospital length of stay, *MODS* multiple organ dysfunction syndrome, *ARDS* adult respiratory distress syndrome

*Statistically significant

^a^1 unit of platelets contains 5 donors

Thirty-eight (16%) patients died; twenty-seven (71%) of them died of TBI, 4 (11%) died of respiratory insufficiency, 2 (5%) died of ischemia after entrapment of the body, 2 (5%) died of cardiac origin, 1 (3%) due to Multiple Organ Dysfunction Syndrome (MODS), 1 (3%) due to hypoxia, and 1(3%) due to sepsis. Median time to death was 11 (6–17) days.

### Outcome

Patients who died were older (68 vs. 39 years, *p* < 0.001) and more severely injured (ISS 38 vs. 30, *P* = 0.02), mainly to the brain and chest. On arrival in ED, they suffered from more severe respiratory acidosis (pH 7.26 vs. 7.30 (*p* = 0.047), PaCO_2_ 48 vs. 44 mmHg (*p* = 0.04), BD − 6.0 vs. − 4.0 mEq/L (*p* = 0.08)), and were more coagulopathic (Prothrombin Time (PT) 14.8 vs. 14.2 s, *p* = 0.03). They received more crystalloids < 24 h (11.3 vs. 8.3 L, *p* = 0.003), but similar number of blood products < 24 h compared to patients who survived. Further, patients who later died developed more often MODS (42 vs 13%, *p* < 0.001), and stayed longer on the ventilator (9 vs. 6 days, *p* = 0.002, Table 1).

**Fig. 2:**
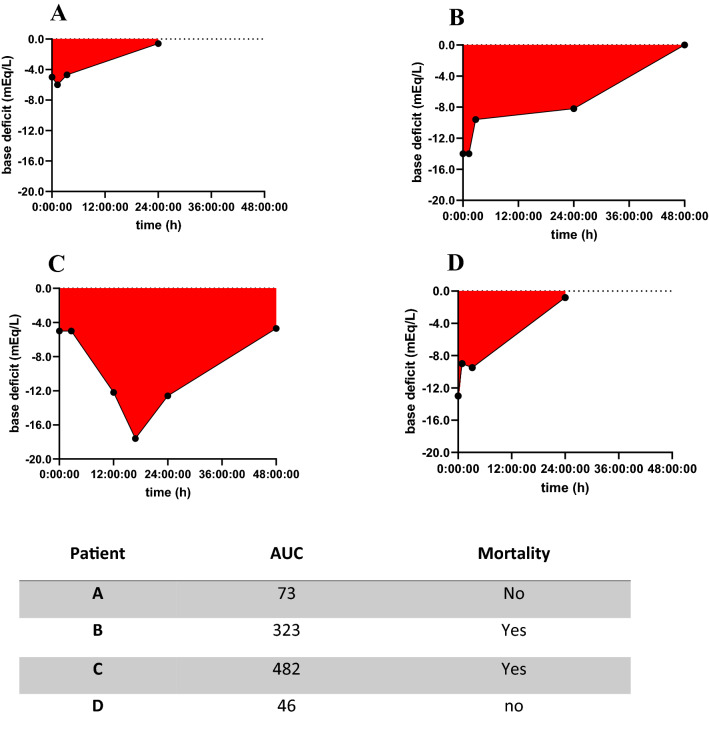
Examples of base deficit in time and area under the curve (AUC-colored area) related to mortality

Additionally, no difference was shown between the single base deficit measurements in ED, OR nor ICU between patients who later died and those who did not. However, patients who later died were more acidotic at 24 h and 48 h after arrival (Table 2). They had also a larger AUC (213 vs. 111, *p* < 0.001) than patients who survived (Table 2). There was no difference in AUC between different causes of death. In Fig. 2 examples of base deficit in in time with calculated AUC (colored area) are shown.

**Table 2:** Separate base deficit (BD) measurements related to mortality

	Survival (*n* = 197)	Mortality (*n* = 38)	*P* value
BD_ED (mEq/L)	− 4.0 (− 7.0–2.0)	− 6.0 (− 9.0–2.3)	0.08
BD_OR (mEq/L)	− 6.0 (− 8.0–3.0)	− 7.0 (− 10.0–4.0)	0.13
BD_ICU (mEq/L)	− 4.6 (− 6.8–2.8)	− 5.2 (− 9.2–3.5)	0.11
BD_24h(mEq/L)	− 2.0 (− 4.3–0.1)	− 4.9 (− 7.5–1.8)	< 0.001*
BD_48h (mEq/L)	0.0 (− 2.0–2.0)	− 1.8 (− 4.8–0.6)	0.001*
AUC	111 (53–192)	213 (114–316)	< 0.001*

Data are expressed in median (IQR)

*BD* base deficit, *ED* emergency department, *OR* operation room, *ICU* intensive care unit, *AUC* area under the curve

*Statistically significant

### Area under the curve

**Fig. 3:**
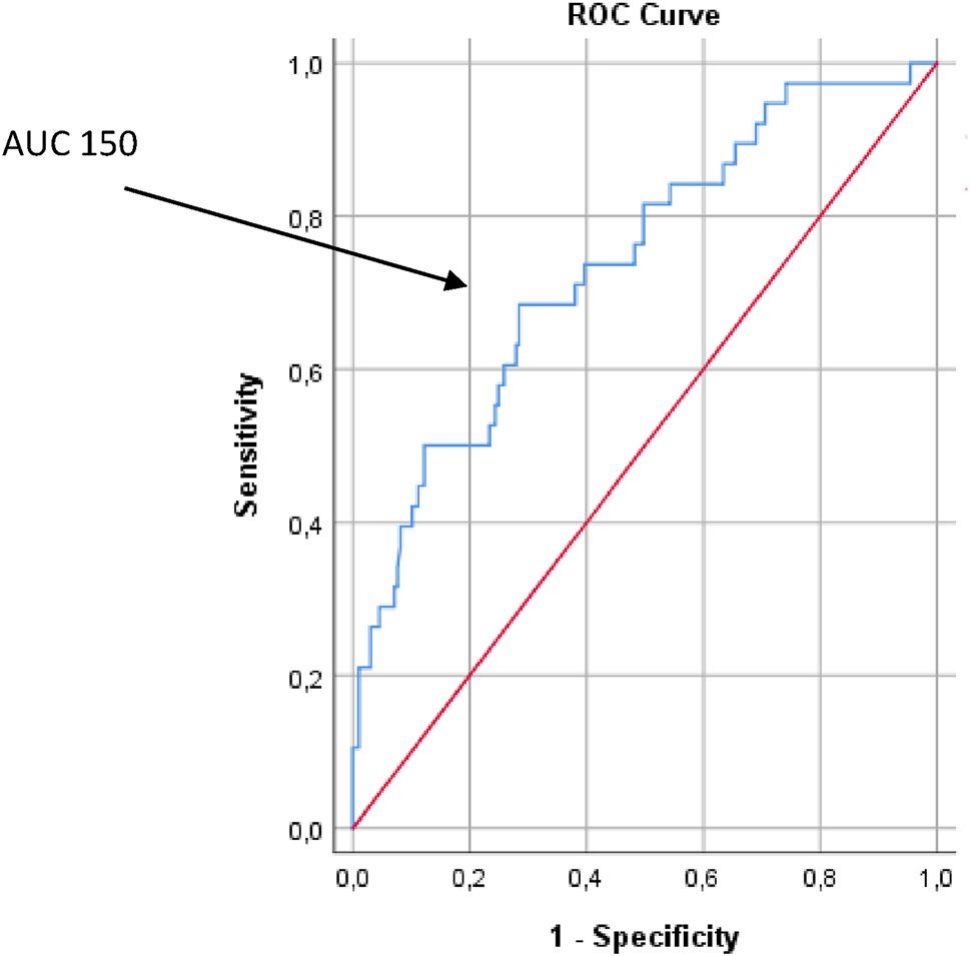
Receiver-operator characteristic (ROC) curve of all included patients

Figure 3 shows the receiver-operator characteristic (ROC) curves of AUC in relation to mortality (AUC 0.74). When AUC > 150 was chosen as cut-off point sensitivity and specificity for mortality were respectively 74 and 38% (Table 3). Further, patients had a 4.7 times higher chance of dying compared to patients with an AUC < 150 (Odds Ratio 4.7 (95% CI 2.1–10.1, *p* < 0.001)).

**Table 3:** Area under the curve (AUC) cut-off 150 related to mortality

	Survival (*n* = 197)	Mortality (*n* = 38)	Total
AUC < 150	74	10	84
AUC > 150	123	28	151
Total	197	38	235

Data are expressed in absolute numbers

Multivariate analysis (included parameters were age, AIShead, crystalloids < 24 h, and AUC) showed that age, AIShead, crystalloids < 24 h, and AUC were independent predictors for mortality (Table 4).

**Table 4:** Multivariate analysis: mortality

Variable	β-Coefficient	*P* value	Odds ratio	95% C.I	
Step 1^a^				Lower	Upper
AUC	0.009	0.000	1.009	1.005	1.012
Constant	− 3.164	0.000	0.042		
Step 2^b^					
Age	0.046	0.000	1.047	1.023	1.071
AUC	0.008	0.000	1.008	1.005	1.012
Constant	− 5.424	0.000	0.004		
Step 3^c^					
Age	0.048	0.000	1.050	1.025	1.074
AUC	0.009	0.000	1.009	1.005	1.013
AIShead	0.341	0.008	1.406	1.095	1.806
Constant	− 6.594	0.000	0.001		
Step 4^d^					
Age	0.052	0.000	1.053	1.028	1.079
AIShead	0.345	0.008	1.412	1.096	1.818
Crystalloids < 24 h	0.000	0.048	1.000	1.000	1.000
AUC	0.007	0.001	1.007	1.003	1.011
Constant	− 7.706	0.000	0.000		

^a^Variable entered on step 1: AUC

^b^Variable entered on step 2: Age

^c^Variable entered on step 3: AIShead

^d^Variable entered on step 4: crystalloids < 24 h

95% *CI* confidence interval, *ISS* injury severity score, *AUC* area under the curve

### Late mortality

There were 27 patients (71%) who died > 7 days after hospital admission. There was no difference in cause of death between early (3–6 days) and late deaths (> 7 days, *p* = 0.38).

Analysis in this subgroup of patients revealed no difference in separate base deficit in ED, OR and ICU compared to surviving patients. Patients who later died had deeper base deficits at 24 h and 48 h. AUC was more than twice as large in patients who later died (231 vs. 111, *p* < 0.001). There was no difference in base deficit during any point in 48 h in patients who died early or late (supplemental Table S1). There was also no difference in AUC regarding cause of death. AUC was also an independent predictor for late mortality.

### Damage control surgery

All patients had surgery within 24 h after arrival in hospital, 167 patients (71%) had a second surgical procedure within 48 h. Fifty-two percent (*n* = 121) of patients had their first surgical procedures performed in damage control setting. DCS patients had a deeper BD on arrival in ED, in OR, and on arrival in ICU than no-DCS patients. However, there was no difference in BD anymore after 24 h and 48 h. There was no difference in AUC between DCS and no-DCS patients (Table 5). Additionally, there was no difference in mortality between patients who underwent DCS and patients who did not (12% (15/121) vs. 20% (23/114), *p* = 0.11), nor was there any difference in time of death; 73% (11/15) of DCS patients and 70% (16/23) of no-DCS patients died > 7 days after admission (*p* = 1.0).

**Table 5:** separate base deficit (BD) measurements related to patients who had DCS and patients who had no DCS

	DCS (*n* = 121)	No-DCS (*n* = 114)	*P* value
BD_ED (mEq/L)	− 5.0 (− 9.0–3.0)	− 3.0 (− 5.0–1.0)	< 0.001*
BD_OR (mEq/L)	− 7.0 (− 12.0–4.0)	− 4.6 (− 7.0–2.0)	< 0.001*
BD_ICU (mEq/L)	− 5.0 (− 7.4–3.4)	− 4.1 (− 6.6–2.6)	0.03*
BD_24h (mEq/L)	−2.2 (− 5.4–0 .0)	– 2.4 (− 5.0–0.8)	0.43
BD_48h (mEq/L)	0.0 (− 2.0–1.9)	− 0.2 (− 2.8–1.8)	0.34
AUC	135 (59–211)	123 (58–202)	0.85

Data are expressed in median (IQR)

*BD* base deficit, *DCS* damage control surgery, *ED* emergency department, *OR* operation room, *ICU *intensive care unit, AUC area under the curve

*Statistically significant

## Discussion

In this study, it was demonstrated that severely injured patients with deranged physiology on arrival in ED died less frequently, if they had a prompt restoration of physiological derangement in the first 48 h after injury. This was both true for early and late deaths. The chance of dying increased four- to fivefold with a large AUC of base deficit in time.

There are only a few reported studies in which serial base deficits were measured in relation to mortality [12, 19]. In this study individual base deficit measurements did not differ between patients who later died and the ones who did not until 24 h after injury. This suggests that the time period in which physiology was restored, was even more important than the early individual base deficit measurements, making AUC an accurate tool for mortality since this includes the time period in which physiology was restored.

This study demonstrated that blood pressure was not a good predictor for identifying patients who are at risk of dying. This is in line with others who have shown that that baseline vital signs such as blood pressure had no correlation with hypoperfusion (lactate and BD), and mortality [7–10]. In literature, there are several reports on the predictive value of lactate [24–27] and base deficit [11–19] as predictor for mortality. There are also a few studies in which serum lactate and base deficit were compared [17, 26, 27]. Some studies [26, 27] found that lactate was a better predictor for death while others showed that, although admission BD and lactate levels are correlated following injury, BD was superior to lactate in identifying shock, need for resuscitation and mortality in polytrauma [11]. In this study, BD was chosen as marker for acidosis since BD was routinely measured in ED, OR, and ICU whereas serum lactate was only measured occasionally. Interestingly, in this study individual base deficit measurements did not differ between patients who later died and the ones who did not until 24 h after injury. One could argue that this could be influenced by the fact that more than half the patients underwent DCS. Even though DCS patients were physiologically more deranged in the early hours after injury, there was no difference in BD anymore after 24 h. There was also no difference in mortality and time of death between patients who underwent DCS and patients who did not. This suggests that the indication for damage control surgery was adequate, and is in line with results from a previous study from our institution [28].

The vast majority of patients in this study died of TBI. This is in line with our previous reports and a global review on mortality rates [1–6]. This large portion of TBI-related deaths is also partially caused by the inclusion criteria since patients who died within 48 h after injury were excluded, thereby excluding death by exsanguination. However, in a previous study in which all in-hospital deaths were included, only a small proportion of our severely injured patients (< 5%) died of hemorrhage [1].

In this study, severely injured patients who had urgent surgery < 24 h (52% of the first surgical procedures were performed in damage control setting) were included in order to select the most physiologically deranged patients. The group of (sub)acute (< 48 h) deaths was excluded since it has been long known that base deficit in early phase after injury is related to early mortality [12, 14, 17]. Less is known about the influence of timely physiology restoration on late deaths in severely injured, especially when TBI is the main cause of death. In this study, it was demonstrated that early restoration of physiology in polytrauma patients is also important for deaths due to TBI later during hospital stay.

In this study, age was an independent predictor for mortality. This could be expected since elderly patients have less physiological reserve and are more likely to be coagulopathic based on the medication they frequently use. Others have described the influence of age on mortality as well [29–31]. Further, patients who later died were more severely injured to the brain and chest, and had a more severe respiratory acidosis on arrival in ED even though there was no difference in prehospital intubation rate. This leaves room for improvement in controlling respiratory acidosis early after injury especially in a patient population in which TBI is the main cause of death.

The amount of crystalloids was large in both groups, although it should be taken into account that this included all crystalloids that were administered within 24 h, including those prehospitally, in ED, ICU and during surgical procedures. Deceased patients received more crystalloids < 24 h than patients who survived. One could argue that patients who received more crystalloids < 24 h had a deeper metabolic acidosis (causing a larger AUC) at 24 h because of the administered crystalloids, and would, therefore, die more often. This is confirmed by the fact that crystalloids < 24 h were an independent predictor for mortality. Additionally, we have demonstrated in a previous study that large amounts of crystalloids are known to attribute to increased mortality [32]. Crystalloid administration < 24 h does not fully explain the differences in AUC between surviving and deceased patients since AUC was measured until base deficit normalization or up to 48 h, and BD was again within normal limits < 48 h in both groups.

To our knowledge, this is the first study in which the time of physiological restoration based on serial BD measurements in the first 48 h in severely injured patients could be related to mortality at least a week later. Based on data from this study, the risk of dying during hospital stay could be estimated a median of 9 days prior to the actual time of death, leaving an opportunity for death prevention. Future studies should focus on strategies to early (< 48 h) normalization of physiology; an effort should be made to control acidosis, including respiratory acidosis by controlling PaCO_2_, in the early days after injury and limit the amount of crystalloid resuscitation. There should be an extra emphasis on preventing secondary damage to the brain since TBI was the main cause of death.

A few limitations need to be acknowledged: first, this was a retrospective analysis of a single-center prospective cohort study with its accompanying limits. Further, treating clinicians were also the researchers. Additionally, base deficit measurements were not corrected for ethanol levels. Another limitation is that no details on comorbidities nor medication were collected.

In conclusion, in this study timing and duration of physiological correction in the first 48 h was associated with both early and late mortality. This highlights the importance of aggressive correction of physiological derangement, which could possibly attribute to a further attenuation of mortality in a population of severely injured patients whose main cause of death was TBI.

### Supplementary Information

Below is the link to the electronic supplementary material.

Supplementary file1 (DOCX 14 KB)

## Data Availability

The dataset supporting the conclusions of this article are available upon reasonable request from the corresponding author.
