# Supplementary material for: Early correction of base deficit decreases late mortality in polytrauma
Source: Eur J Trauma Emerg Surg. 2022 Nov 22;50(1):121–9. doi: 10.1007/s00068-022-02174-9 (PMC10924017; doi:10.1007/s00068-022-02174-9)
Supplement: Supplementary file 1 — Supplementary file1 (DOCX 14 KB) [file 68_2022_2174_MOESM1_ESM.docx]

**Table S1.** separate base deficit (BD) measurements related to time of death

|  | Early death  (n=11) | Late deaths  (n=27) | P-value |
| --- | --- | --- | --- |
| BD_ED (mEq/L) | -8.0 (-12.8—3.3) | -4.5 (-8.3—2.0) | 0.20 |
| BD_OR (mEq/L) | -8.0 (-12.0—4.0) | -7.0 (-9.0—4.0) | 0.39 |
| BD_ICU (mEq/L) | -6.9 (-10.1—5.2) | -4.1 (-7.0—3.1) | 0.08 |
| BD_24h (mEq/L) | -4.1 (-7.0—2.0) | --5.7 (-8.5—2.3) | 0.25 |
| BD_48h (mEq/L) | -5,1 (-8.4-1.0) | -1.7 (-3.3-0.3) | 0.41 |
| AUC | 193 (149-358) | 231 (114-314) | 0.90 |

BD=base deficit, DCS=damage control surgery, ED=emergency department, OR= operation room, ICU=intensive care unit, AUC=area under the curve

Data are expressed in median (IQR)

*statistically significant
